# Syringomatous Adenoma of the Nipple: A Rare Benign Breast Tumor

**DOI:** 10.7759/cureus.107686

**Published:** 2026-04-25

**Authors:** George Simatos, Christos Kalyvopoulos, Aristofania Simatou, Ilianna Kougia, Eleni Papaioannou

**Affiliations:** 1 First Department of Breast Surgery, Anti-Cancer Hospital of “Saint Savvas”, Athens, GRC; 2 Second Department of Oncology, Anti-Cancer Hospital of “Saint Savvas”, Athens, GRC; 3 Fifth Department of Breast Surgery, Mitera Hospital, Athens, GRC; 4 Department of Pathology, University General Hospital of Larissa, Larissa, GRC

**Keywords:** benign breast condition, benign breast disease, breast disease, breast surgery, syringomatous adenoma of nipple

## Abstract

Syringomatous adenoma of the nipple (SAN) is a rare benign tumor of the breast that can become a clinical and diagnostic challenge, as it can be misdiagnosed as invasive carcinoma due to its infiltrative nature. The treatment of choice is wide resection with clear margins. There are limited published cases of SAN.

We present a case of SAN in a 42-year-old female patient, with regard to clinical manifestations, histopathologic and surgical findings, and differential diagnosis from other entities. A literature review of histologically confirmed cases of SAN revealed a total of 61 cases, including ours.

## Introduction

Syringomatous adenoma of the nipple (SAN) is a rare benign adnexal tumor of the breast that poses a significant clinical and diagnostic challenge due to its locally infiltrative growth pattern and its resemblance to invasive carcinoma. It is believed to arise from eccrine structures of the nipple-areolar complex or from multipotential adnexal keratinocytes capable of both follicular and sweat gland differentiation [1,2].

SAN belongs to the broader spectrum of adnexal neoplasms, which includes both benign and malignant entities such as microcystic adnexal carcinoma. These lesions share overlapping histopathological features, including infiltrative growth and ductal differentiation, which may complicate diagnosis. Despite its locally aggressive behavior, SAN is a benign tumor with no metastatic potential; however, misdiagnosis may lead to overtreatment.

To date, only a limited number of cases have been reported in the literature, highlighting the rarity of this entity. In our review, a total of 61 histologically confirmed cases were identified, including the present case.

We aim to present a case of SAN, emphasizing its clinical presentation, diagnostic challenges, histopathological features, and surgical management, along with a focused review of the literature.

## Case presentation

A 42-year-old premenopausal female presented with a five-month history of progressive thickening of the left nipple and areola. Her gynecological history was unremarkable, apart from two previous cesarean sections. There was no history of hormonal therapy, trauma, or significant breast disease.

On clinical examination, a firm retroareolar mass, measuring approximately 3 cm, was palpated in continuity with the nipple-areolar complex. The lesion was associated with localized thickening but without skin ulceration or nipple discharge. No axillary lymphadenopathy was detected. The clinical appearance raised suspicion for Paget’s disease of the nipple.

**Figure 1:**
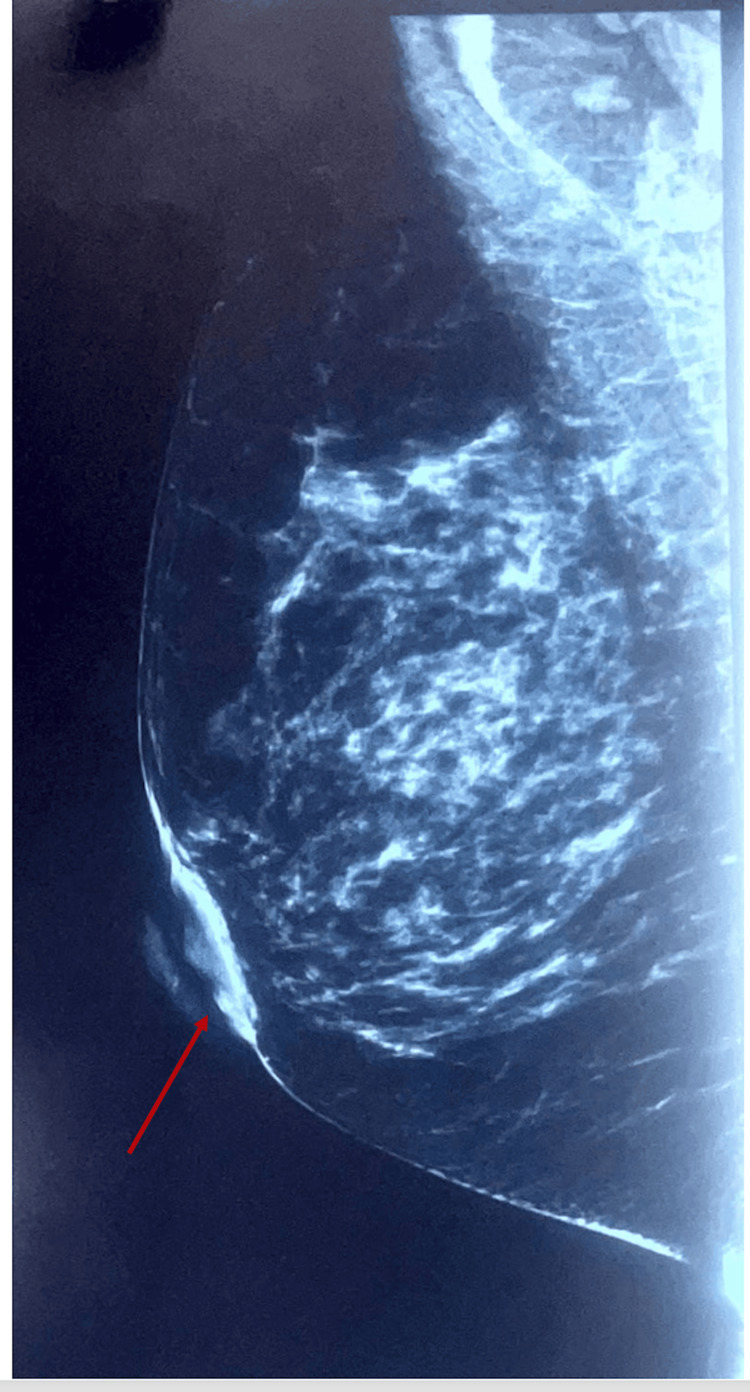
Preoperative mammogram showing retroareolar thickening of the breast (arrow indicating the area of interest).

Mammography demonstrated retroareolar thickening without a clearly defined mass (Figure 1). Ultrasound evaluation did not reveal additional abnormalities. Fine needle aspiration (FNA) cytology was performed preoperatively and was negative for malignancy.

**Figure 2:**
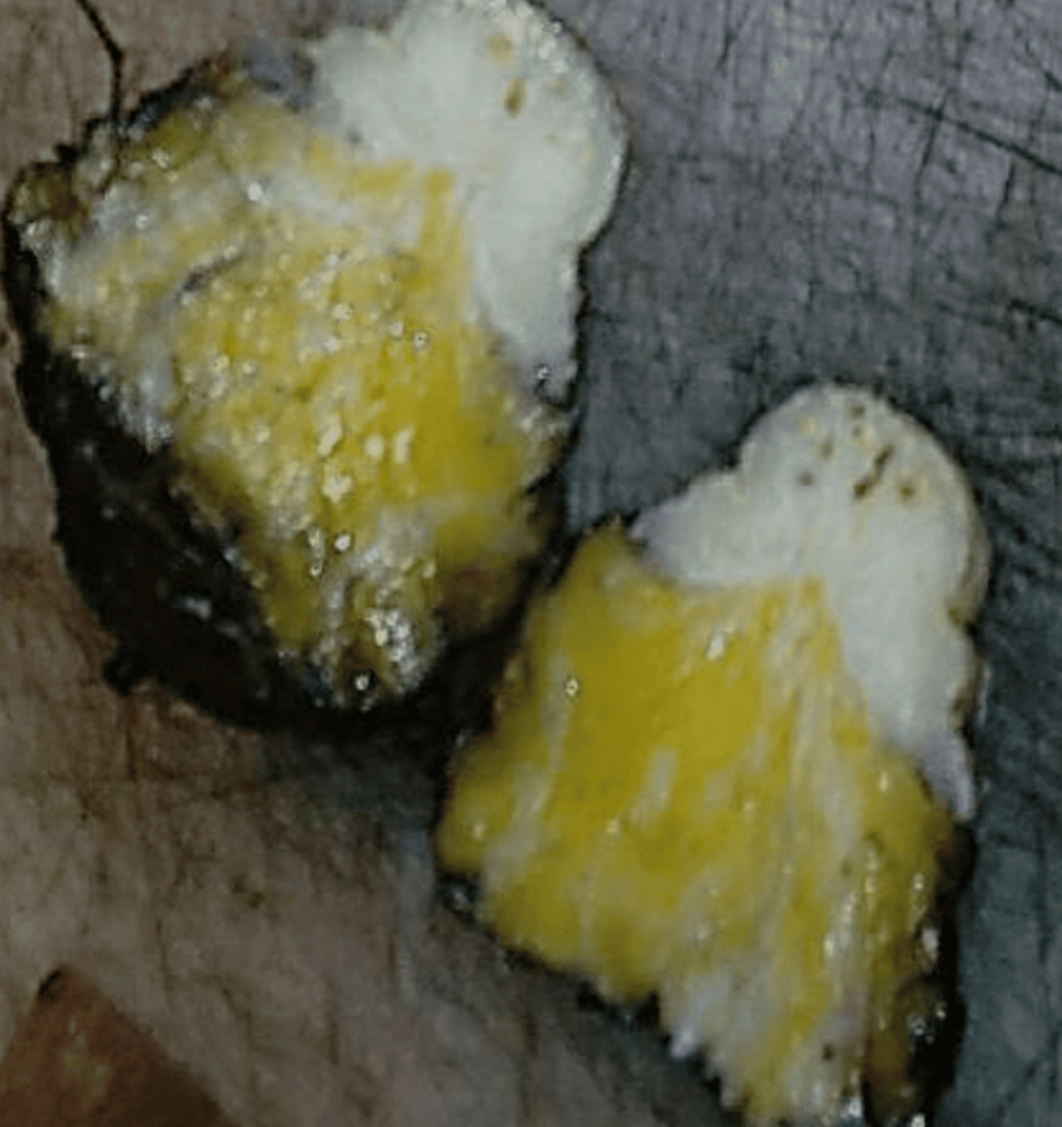
Protruded areola with a knob-like configuration and whitish appearance. On dissection, clear margins with a microcystic appearance were observed.

The patient underwent an excisional biopsy, including part of the nipple-areolar complex (Figure 2). Histopathological analysis suggested SAN. Subsequently, a wider excision was performed, including the nipple and areola, ensuring clear surgical margins.

On macroscopic examination, the specimen demonstrated a thickened areola with a nodular, whitish appearance. On cross-section, a fibroelastic lesion with focal microcystic areas was identified. Surgical margins, as assessed with ink, were free of tumor.

**Figure 3:**
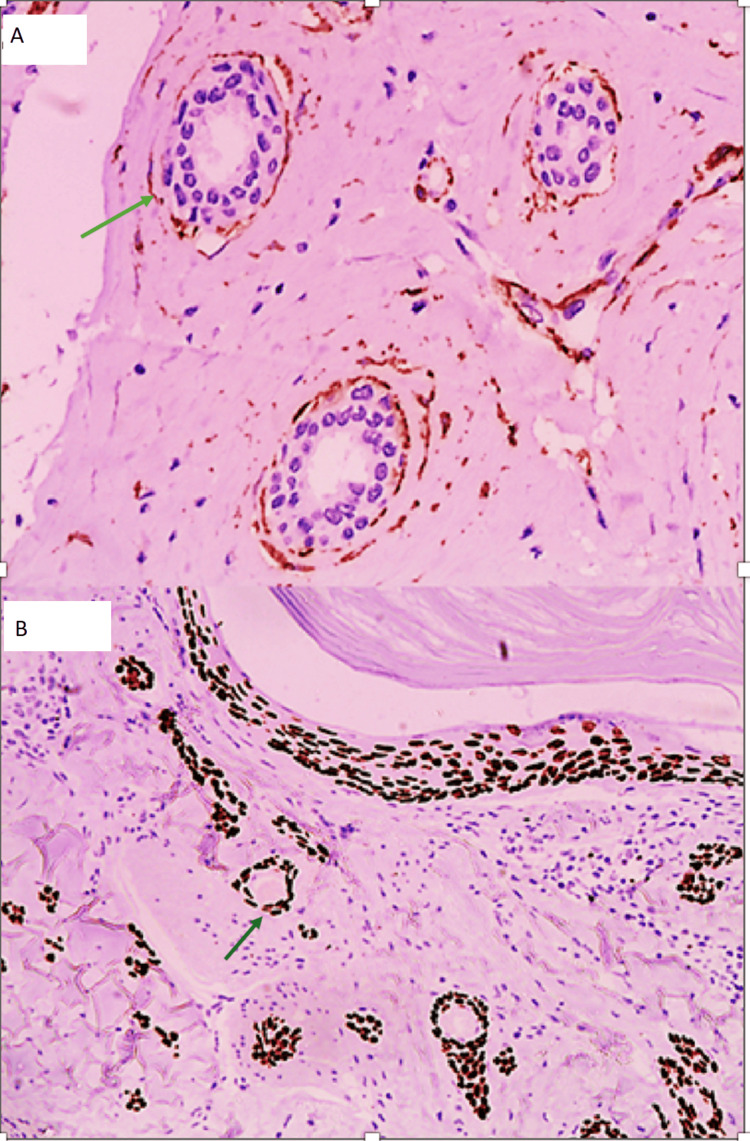
Immunohistochemical staining showing positivity for smooth muscle actin (SMA) and p63. (A) Smooth muscle actin (SMA) stains the entire perimeter of the myoepithelial cells of the neoplastic glands in a double layer, suggesting a benign condition (arrow indicating double layer). (B) p63 stains the outer layer of myoepithelial cells, suggesting a double layer (benign condition) (arrow indicating double layer).

Immunohistochemical staining showed positivity for p63 and smooth muscle actin (SMA), confirming the presence of myoepithelial cells and supporting a benign diagnosis (Figures 3A-3B). The tumor was negative for estrogen and progesterone receptors.

**Figure 4:**
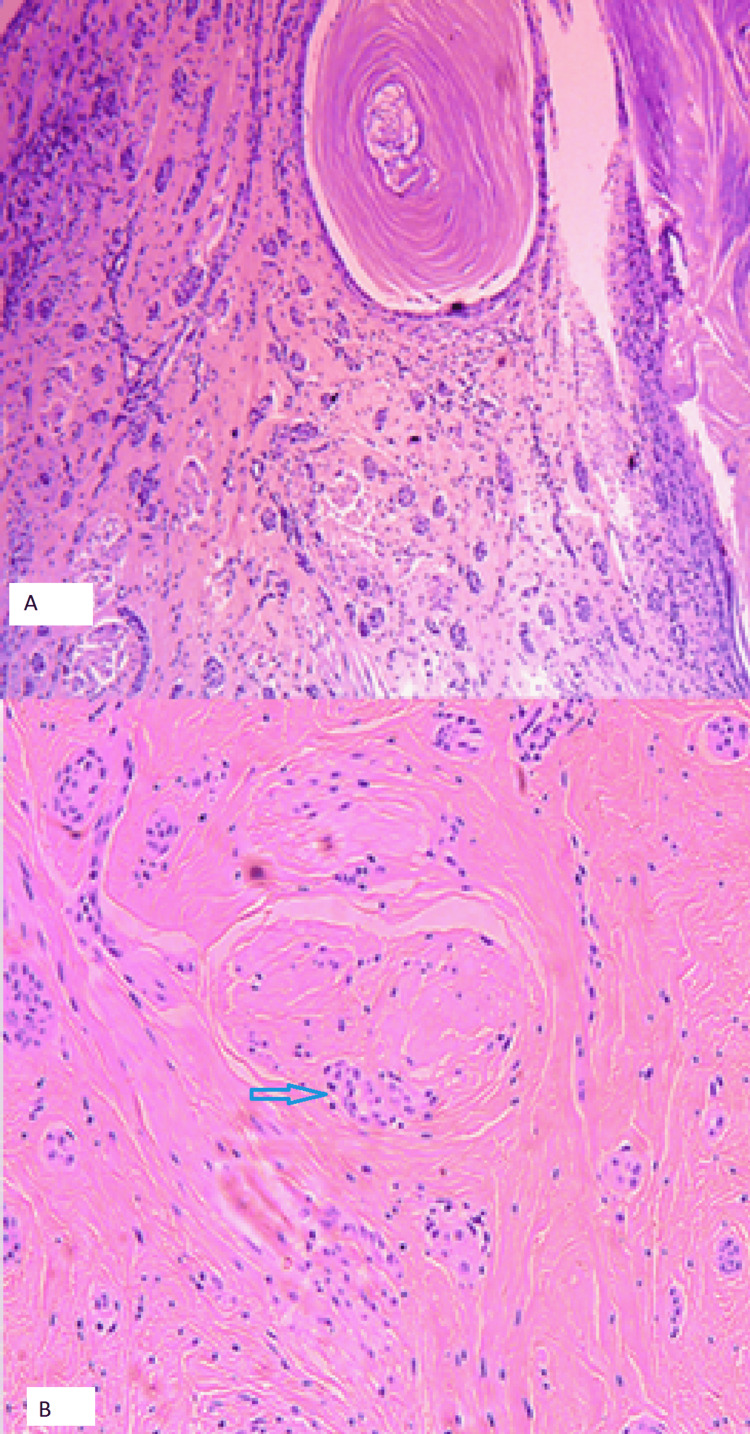
Neoplasm with a tubular and infiltrative pattern among lactiferous ducts and muscular fibers (A), and foci of perineural proliferation (B). Histopathological features of syringomatous adenoma of the nipple. (A) Hematoxylin and eosin (H&E) staining demonstrating infiltrative ductal and tubular structures embedded within a fibrous stroma (H&E stain; original magnification ×100), with adjacent keratin cyst formation. (B) Higher magnification view showing infiltrating glandular structures within desmoplastic stroma (H&E stain; original magnification ×200). The arrow highlights a small duct-like structure lined by epithelial cells, consistent with the characteristic infiltrative growth pattern.

Histological examination revealed an infiltrative neoplasm composed of small tubular and duct-like structures embedded within a fibrous stroma, extending between lactiferous ducts and smooth muscle fibers. Foci of squamous differentiation and perineural proliferation were identified. The lesion demonstrated a characteristic, double-layered architecture consisting of luminal epithelial cells and an outer myoepithelial layer (Figure 4).

The patient declined nipple reconstruction. She remains under annual follow-up and is asymptomatic, with no evidence of recurrence at 54 months.

## Discussion

SAN is a rare benign adnexal tumor first described by Rosen in 1983 [1]. It is characterized by locally infiltrative growth and a tendency to mimic malignant breast lesions both clinically and histologically, which may lead to misdiagnosis and overtreatment. Despite its infiltrative nature, SAN has no metastatic potential, and lymph node involvement has not been reported [1].

In a review of the literature in 2009, Oo and Xiao identified 34 cases of SAN, including 32 female and 2 male patients, with a mean age at presentation of 46.1 years [3]. Since then, additional cases have been reported. Park et al. identified a further 18 cases up to March 2022 [4]. In our literature review, including previously published reports and additional identified cases [5-13], a total of 61 histologically confirmed cases were found. Among these, rare presentations included bilateral lesions [13,14], occurrence in supernumerary nipples [8], and cases in male patients [15,16]. A small number of cases have been reported in association with pregnancy and lactation, suggesting a possible hormonal influence [5].

Clinically, SAN typically presents as a solitary retroareolar mass, often associated with nipple thickening, inversion, or discharge. In some cases, the presentation may mimic Paget’s disease or carcinoma. Imaging findings are non-specific. Mammography may demonstrate a high-density retroareolar lesion with irregular margins and occasional microcalcifications, while ultrasound findings may include a hyperechoic or heterogeneous mass with indistinct borders [4]. These features are not diagnostic and often necessitate histopathological confirmation.

SAN belongs to the spectrum of adnexal tumors with follicular and sweat gland differentiation, which includes both benign lesions and malignant counterparts such as microcystic adnexal carcinoma. These entities share overlapping histological features, including infiltrative growth and ductal differentiation, making accurate diagnosis essential [3,4]. Imaging findings are often non-specific, typically demonstrating irregular retroareolar thickening or a poorly defined mass on mammography and ultrasound [4]. Histopathological evaluation remains the cornerstone of diagnosis. The presence of a dual-cell layer with preservation of myoepithelial cells, confirmed by markers such as p63 and SMA, is critical in distinguishing SAN from malignant mimics such as tubular carcinoma and low-grade adenosquamous carcinoma, in which myoepithelial cells are absent [3,4,9]. Complete surgical excision with clear margins remains the treatment of choice, as incomplete resection is associated with a significant risk of local recurrence [3].

Histologically, SAN is characterized by small infiltrating ducts and tubules embedded within a fibrous stroma, often extending between lactiferous ducts and smooth muscle bundles [3,4]. The presence of squamous differentiation and keratin cyst formation is a distinguishing feature [3]. Perineural invasion, although typically associated with malignancy, may also be observed in SAN and should not be misinterpreted as a sign of aggressive behavior [3,4]. The defining feature remains the preservation of a double-layered structure composed of luminal epithelial and myoepithelial cells. Immunohistochemical staining plays a crucial role in confirming this architecture, with markers such as p63 and SMA highlighting the myoepithelial layer and supporting a benign diagnosis [4,9]. In contrast, malignant lesions such as tubular carcinoma lack a myoepithelial component and typically express hormone receptors [3].

The differential diagnosis includes adenoma of the nipple, tubular carcinoma, and low-grade adenosquamous carcinoma [3,4]. Adenoma of the nipple is usually a well-circumscribed lesion with less infiltrative behavior [3]. Tubular carcinoma is characterized by a single layer of epithelial cells without myoepithelial support and typically demonstrates estrogen receptor positivity [3]. Low-grade adenosquamous carcinoma may exhibit infiltrative growth and squamous differentiation but shows cytological atypia and lacks the well-organized dual-layered structure seen in SAN [4].

Due to its rarity and diagnostic ambiguity, management strategies for SAN have varied historically, ranging from local excision to mastectomy with lymph node dissection [15]. This variability reflects the difficulty in distinguishing SAN from malignant lesions preoperatively. Current evidence supports complete surgical excision with histologically clear margins as the optimal treatment. In cases where the lesion involves the dermis and subcutis of the nipple-areolar complex, excision of the nipple and areola is recommended. In selected cases where involvement is limited, nipple-sparing excision with oncoplastic reconstruction may be considered, particularly in younger patients [13].

Incomplete excision has been associated with recurrence rates as high as 45% in some series [3], although these cases were primarily related to inadequate initial resection. Recurrence has been reported within 1.5 to 4 years following incomplete excision [4]. In contrast, no recurrences have been reported among patients with clear surgical margins during follow-up periods of one to six years [13]. Therefore, long-term follow-up of at least five years is recommended.

In conclusion, SAN is a rare benign but locally infiltrative tumor that can closely mimic malignancy. Accurate diagnosis relies on careful histopathological and immunohistochemical evaluation. Awareness of this entity is essential to avoid overtreatment. Complete surgical excision with clear margins remains the cornerstone of management, with excellent long-term outcomes when appropriately treated.

## Conclusions

SAN is a rare, benign, locally infiltrating tumor. As misdiagnosis with tubular carcinoma or adenosquamous carcinoma is possible, high clinical suspicion and appropriate histopathologic examination are essential to establish the diagnosis.

The optimal management is resection with free margins. If the dermis and subcutis of the areola are free, a nipple-sparing resection with oncoplasty is the treatment of choice. If the nipple and areola are affected, resection of them should be included, with the possibility of delayed reconstruction as an option. Careful monitoring is essential for a period of at least five years.
